# Molecular genetics to genomic medicine practice: at the heart of value-based delivery of healthcare

**DOI:** 10.1002/mgg3.8

**Published:** 2013-05-15

**Authors:** Charis Eng

**Affiliations:** 1 Genomic Medicine Institute, Lerner Research Institute, Taussig Cancer Institute and Stanley Shalom Zielony Institute of Nursing Research, Cleveland ClinicCleveland, OH, 44195; 2 Department of Genetics and Genome Sciences, CASE Comprehensive Cancer Center, Case Western Reserve University School of MedicineCleveland, OH, 44116

Knowledge is of no value unless you put it into practice.

Attr. Heber Grant, ca 1945

The current prevailing model of healthcare delivery in the United States is volume and fee-for-service. Those days are close to over. Value-based healthcare delivery is the watchword of the day, intuitively well meaning and well received, and yet, fear lurks not far behind for both health caregivers and patients. The practice of genetics has never been volume driven nor fee driven: genetics professionals have always delivered and will continue to deliver value-based healthcare.

Value-based healthcare delivery is the “in” phrase in all of clinical practice, not only in the United States, but also internationally (Elshaug et al. 2013). In the healthcare field, value broadly means the most effective clinical maneuvers that provide the best benefit for the patient in a cost-efficient manner (Porter and Teisberg 2006). The simple-minded amongst us, like myself, would think that research evidence-based clinical practice would automatically lead to value in healthcare.

Effective research minimally leads to the acquisition of fundamental scientific knowledge. Biomedical researchers, health caregivers, and the general public alike all want the transference of rigorously acquired knowledge to the practice of evidence-based medicine. The translation of molecular genetics and genomic medicine knowledge to the practice of genomic medicine appear an ideal, seemingly straightforward, paradigm for this type of rapid translation of knowledge to value-based clinical practice. After all, what is more clear than finding a genetic alteration which leads to an accurate molecular diagnosis and a genotype-enabled management path resulting in early detection and/or prevention?

The above sentiment is reflected in the U.S. Department of Health and Human Services' recently released Healthy People 2020 benchmarks aimed at improving the healthcare of all Americans and target the two most common cancers where fundamental basic, translational, and clinical investigation over a decade to a decade and a half have suggested clinical utility and actionability. In virtually every developed country of the world, government-led efforts for healthier citizens has become routine. For instance, every decade, the United States Department of Health and Human Services releases public health-directed goals for the general population, referred to as “Healthy People xx” where xx represents the decade. These healthcare objectives are released every decade using firm evidence-based information regarding cost-effective clinical benefits at the population level. The immediate past set of national goals set in 2000, Healthy People 2010, included eating a balanced meal comprising all the food groups and regular exercise. Never before has genetics or family health histories played a role in these goals. The 2020 objectives included new health promotion areas to concentrate on and for the first time include genomic medicine in the list of priorities. The genomic objectives of Healthy People 2020 emphasize the importance of obtaining a family and genetic history as a potential and powerful guide for clinical and public health initiatives. The first genomic recommendation is that women with a family history of breast or ovarian cancer should receive genetic counseling. In 2005, 23% of women with a family history of breast and/or ovarian cancer received genetic counseling. In a 2009 nation-wide study, this figure is even lower, 4% (Levy et al. 2009). Healthy People 2020 seeks to increase this to 26% or at least a 10% improvement (U.S. Department of Health and Human Services 2010). The second recommendation is to increase the number of patients newly diagnosed with colorectal cancer that obtain genetic testing to rule out Lynch syndrome. These genomic recommendations are based on the thought that knowing this information will lead to gene-enabled management and improve the health of involved patients. By increasing one's knowledge through genetic counseling and appropriate testing, the risk of developing cancer in high-risk individuals can be significantly reduced.

While there are many examples of genetics and genomic medicine's value in healthcare, one of the best illustrations is Lynch syndrome, an autosomal dominant disorder and the most common adult-onset inherited colorectal cancer syndrome, occurring in one in 35 individuals presenting with colorectal cancer. Lynch syndrome is caused by germline mutations in one of several mismatch repair genes, *MLH1, MSH2,* and *MSH6*, and more rarely, *PMS2*. In addition to an up to 85% lifetime risk of colorectal cancer, Lynch syndrome confers a 40% lifetime risk of endometrial cancer, and 10% ovarian cancer as well as elevated risks of gastric and hepatobiliary cancers. Early and regular surveillance has been shown to save lives from colorectal cancer. A cellular phenotype of mismatch repair deficiency is microsatellite instability (MSI). Conveniently, germline mutation in a mismatch repair gene manifests as lack of expression of the corresponding mismatch repair protein which can be easily visualized by immunohistochemical examination [IHC] (Aaltonen et al. 1998). Such molecular genetic evidence easily reveals a cost-effective manner to screen all colorectal cancers. So far, so good!

In traditional biomedical parlance, the valley of death refers to the huge gulf between fundamental discovery, typically of a new drug target and its associated basic science research, and the approval of a drug for clinical use (Hudson and Kharzragui 2013). Is the gulf between acquisition of fundamental genomics knowledge and the practice of genomic medicine the 21st century valley of death? As Michael Porter, one of the doyens of value-based healthcare delivery, likes to say, the American healthcare system uses a 1960s infrastructure and has placed multiple patches and band-aids for the last 50 years so that we can limp through 21st century healthcare delivery (Porter and Teisberg 2006). Implementing a very modern type of medicine – genetic and genomic medicine – in a 1960s health system infrastructure could be responsible for this new valley of death. This lies at the heart of the great efforts required for implementation of genomics research to genomic medicine practice, again ably illustrated by Lynch syndrome screening.

While many academic medical centers see the value and cost–benefit of universal MSI/IHC screening of all colorectal and endometrial cancers for Lynch syndrome, they have faced unforeseen barriers, including the inability of their departments of pathology to bring on such testing, for a number of reasons. Many departments of pathology across the country have successfully instituted universal MSI/IHC screening and note the screening results in their pathology report, some as early as 2000 (Hampel et al. 2005). However, on later evaluation of the implementation, virtually all institutions found that only a small proportion of screen positive individuals were referred to genetic professionals (Heald et al. 2013). In our own institution, for example, only half of the screen positive individuals were referred to genetics clinic when relying on the surgeon of record to act on traditional pathology reporting. In contrast, when screen positive results became routinely sent to a dedicated genetic counselor, 100% of screen positive individuals were referred (Heald et al. 2013). From beginning universal Lynch syndrome screening to the final successful approach took 4 years filled with multiple meetings and consensus building, and integrating genetic counselors in the colorectal cancer clinic and high-risk colon cancer clinic. An important lesson learnt in the universal Lynch screening experience, which is generalizable, is that failure of clinical implementation or incomplete/haphazard implementation of perfectly good genomic medicine, based on rigorous molecular genetics evidence, is not the equivalent of “genomic medicine does not work”.

Just in the last decade alone, we have been gifted with rapid derivation of new genomic and multi-omic data and the technology and know-how of how to integrate them. When new toys and new data arrive, there is a very human tendency to embrace “out with the old, in with the new.” The greatest lesson learnt throughout history is that the quickest successes come when we follow the concept of “in with the old, in with the new.” For example, until every single genome-wide variation is absolutely linked to a clear clinical outcome, utilizing family health history to guide the interpretation of genome sequencing would be wise (Doërr and Eng 2012).

The successful implementation of genomic medicine with maximal value requires several elements, ranging from the most rigorous research data in molecular genetics associated with well-annotated phenotype, implementation to clinical practice, and iterative evaluation of the entire process from research-derived data to successful implementation at every step, to the associated ethical, legal, and social issues, not to mention, having or investing in the correct infrastructure. It is also vital to keep in mind that value does not only mean the cheapest: a large and important part of value-based healthcare relies on deriving the greatest benefit for the patient and his/her family as well. I look forward to the Journal as a critical and open outlet for original research, discussion, and perspectives surrounding the broad field of molecular genetics and genomic medicine.

“Primum non nocere” (Above all, do no harm)

Attr. Thomas Sydenham, 1624–1689
